# An automated calculation pipeline for differential pair interaction energies with molecular force fields using the Tinker Molecular Modeling Package

**DOI:** 10.1186/s13321-024-00890-5

**Published:** 2024-08-08

**Authors:** Felix Bänsch, Mirco Daniel, Harald Lanig, Christoph Steinbeck, Achim Zielesny

**Affiliations:** 1 Institute for Bioinformatics and Chemoinformatics, Westphalian University of Applied Sciences, August-Schmidt-Ring 10, 45665 Recklinghausen, Germany; 2 Erlangen National High Performance Computing Center (NHR@FAU), Friedrich-Alexander-Universität Erlangen-Nürnberg (FAU), Martensstraße 1, 91058 Erlangen, Germany; 3 Institute for Inorganic and Analytical Chemistry, Friedrich-Schiller University, Lessing Strasse 8, 07743 Jena, Germany

**Keywords:** Intermolecular interaction, Nonbonding interaction, Molecular force field, Molecular modeling, Molecular dynamics, Monomer, Dimer, Flory–Huggins parameter, Isotropic repulsion, Dissipative particle dynamics, DPD, Non-ionic polyoxyethylene alkyl ether surfactant, C_10_E_4_

## Abstract

An automated pipeline for comprehensive calculation of intermolecular interaction energies based on molecular force-fields using the Tinker molecular modelling package is presented. Starting with non-optimized chemically intuitive monomer structures, the pipeline allows the approximation of global minimum energy monomers and dimers, configuration sampling for various monomer–monomer distances, estimation of coordination numbers by molecular dynamics simulations, and the evaluation of differential pair interaction energies. The latter are used to derive Flory–Huggins parameters and isotropic particle–particle repulsions for Dissipative Particle Dynamics (DPD). The computational results for force fields MM3, MMFF94, OPLS-AA and AMOEBA09 are analyzed with Density Functional Theory (DFT) calculations and DPD simulations for a mixture of the non-ionic polyoxyethylene alkyl ether surfactant C_10_E_4_ with water to demonstrate the usefulness of the approach.

**Scientific Contribution**

To our knowledge, there is currently no open computational pipeline for differential pair interaction energies at all. This work aims to contribute an (at least academically available, open) approach based on molecular force fields that provides a robust and efficient computational scheme for their automated calculation for small to medium-sized (organic) molecular dimers. The usefulness of the proposed new calculation scheme is demonstrated for the generation of mesoscopic particles with their mutual repulsive interactions.

## Introduction

The quantitative description of non-bonding interactions between molecules is fundamental to understanding and designing chemical processes in materials and life sciences [1, 2]. In contrast to covalent bonding within molecules, non-bonding intermolecular interactions comprise dispersed variations of electromagnetic interactions like dipole/dipole, dipole/induced dipole, induced dipole/induced dipole (van der Waals) interactions, hydrogen bonding, (partial) charge interactions, π–π, cation/anion–π or polar π-effects. Each different spatial configuration of two molecules can be assigned a corresponding nonbonding intermolecular interaction energy. Its determination is challenging because the intermolecular interactions are generally small compared to covalent bonding and especially to the total energy of a molecular configuration.

For an accurate quantitative description, a quantum chemical treatment with a suitable model chemistry is commonly advised, e.g., application of Density Functional Theory (DFT) with an appropriate combination of functional and basis set: the complexation energy (i.e., the energy difference between a specific dimer configuration and the two monomer molecules that form it) can then quantify the intermolecular interaction. In particular, Symmetry-Adapted Perturbation Theory (SAPT) allows direct computation of non-bonding intermolecular interactions (i.e., without the need to calculate the total energies of monomers and dimer) and provides a physically meaningful decomposition of its contributing (electrostatics, induction, dispersion, short-range repulsion) terms [2]. Recent DFT-SAPT approaches have demonstrated a comparatively fast calculation in combination with remarkable accuracy for small- to medium-sized dimers up to the adenine–thymine base pair [3, 4].

When multiple spatial dimer configurations need to be sampled, quantum chemical approaches become increasingly expensive due to their considerable computational complexity, as a single calculation can easily take minutes or even longer. As a purely classical alternative, molecular force fields may be employed instead: they allow intermolecular energy calculations within fractions of a second for a specific spatial dimer configuration, i.e., an acceleration by several orders of magnitude. This comes at the expense of accuracy, as a given force field can easily lead to deceptively erroneous results.

This work aims at providing a robust automated molecular force-field based calculation pipeline for comprehensive estimation of mutual intermolecular energies of a set of small to medium-sized monomer molecules. For a chosen force field, the monomer molecules must be provided with an initial, chemically intuitive spatial geometry with associated atom types (where for small molecules a planar 2D geometry provided by any 2D structure editor seems to be sufficient). Then, each monomer start geometry is globally optimized to its minimum energy conformer. Several monomer–monomer distances with multiple configurations at each distance are sampled to obtain a near-global minimum energy dimer. This dimer is then locally optimized towards its global minimum. The sampled configurations can be averaged by Boltzmann weights to get mean non-bonded dimer interactions at different distances. The calculations can be extended to obtain differential molecule pair interaction energies, which describe the excess intermolecular interaction of two different molecules in comparison to the average interaction of the molecules themselves (see Eqs. 1 and 2 in “Methods” section below), where molecular dynamics (MD) simulations are used to estimate the coordination numbers of neighboring molecules.

The resulting interaction energies may be useful for different purposes. Based on the comprehensive sampling, suitable spatial start configurations can be obtained for more elaborate (e.g., quantum chemical) refinement procedures. Pairwise non-bonded intermolecular interactions can be considered as molecule-pair descriptors for Cheminformatics tasks like molecular similarity estimation. Differential molecule pair interaction energies play an important role in statistical thermodynamics, e.g., for the quantitative estimation of excess quantities that determine the properties of mixtures [5]. In particular, they can be related to Flory–Huggins interaction parameters for polymer models (Eq. 9), which in turn can be used to describe isotropic particle–particle repulsions for mesoscopic simulation approaches (Eq. 10) [6]. The latter application is particularly studied in this work.

The backbone of the calculation pipeline is implemented using the Java programming language. All force-field-based calculations are performed with the Tinker Molecular Modelling package [7]. The Tinker package is modularized into stand-alone, task-based executables (marked in italics in “Methods” section below), which fit well into the Java backbone-driven parallelized computational scheme that fully exploits the computational capabilities of multicore workstations. All force fields provided by Tinker can be used for the calculation pipeline.

## Methods

The force-field-based intermolecular energy ${E}_{ij}^{C}\left(r\right)$ between two molecules $i$ and $j$ is determined by different non-covalent-bonding contributions (van der Waals and partial charge interactions, hydrogen bonding etc.) and depends on the intermolecular distance $r$ between the centers of the molecules as well as their relative spatial configurations $C$. For each specific distance ${r}_{fix}$ there is a minimum energy configuration ${C}_{min}$ with ${E}_{ij}^{{C}_{min}}\left({r}_{fix}\right)\le {E}_{ij}^{C}\left({r}_{fix}\right)$. The global minimum energy dimer is characterized by a distinct distance ${r}_{min}$ so that ${E}_{ij}^{min}={E}_{ij}^{{C}_{min}}\left({r}_{min}\right)\le {E}_{ij}^{C}\left(r\right)$, i.e. ${r}_{min}$ is the distance between the centers of two molecules $i$ and $j$ when the dimer geometry corresponds to the global energy minimum. If different spatial configurations with a fixed intermolecular distance ${r}_{fix}$ are averaged, an averaged intermolecular energy for this distance $\langle {E}_{ij}\rangle \left({r}_{fix}\right)$ is obtained: The corresponding minimum energy distance ${r}_{\langle E\rangle ,min}$ with ${\langle {E}_{ij}\rangle }^{min}=\langle {E}_{ij}\rangle \left({r}_{\langle E\rangle ,min}\right)\le \langle {E}_{ij}\rangle \left(r\right)$ does not necessarily coincide with minimum distance ${r}_{min}$ of the global minimum energy dimer. Among the concrete averaged configurations with a fixed intermolecular distance ${r}_{fix}$ the configuration ${C}^{*}$ with the minimal intermolecular energy is denoted ${E}_{ij}^{{C}^{*}}\left({r}_{fix}\right)\ge {E}_{ij}^{{C}_{min}}\left({r}_{fix}\right)$.

A differential pair interaction energy describes the excess intermolecular interaction of molecules $i$ and $j$ in comparison to the average interaction of the molecules themselves. This may be defined with respect to the global minimum energy dimers

1 $$\Delta E_{ij} = E_{ij}^{min} - \frac{1}{2}\left( {E_{ii}^{min} + E_{jj}^{min} } \right)$$

or corresponding averages (see Eqs. 5 and 6 below for calculation details)

2 $$\Delta \left\langle {E_{ij} } \right\rangle = \left\langle {E_{ij} } \right\rangle^{min} - \frac{1}{2}\left( {\left\langle {E_{ii} } \right\rangle^{min} + \left\langle {E_{jj} } \right\rangle^{min} } \right).$$

A positive (negative) differential pair interaction energy indicates a less (more) favorable intermolecular interaction compared to the individual ones. When differential pair interaction energies are applied to lattice models, all vertices of the lattice have a fixed number of surrounding neighbours (e.g. $Z=4$ in two dimensions or $Z=6$ in three dimensions). In contrast, continuum models can (and usually do) have different coordination numbers ${Z}_{ij}$, where the number ${Z}_{ij}$ of molecules $j$ surrounding molecule $i$ can (and usually does) differ from the number ${Z}_{ji}$ of molecules $i$ that surround molecule $j$. Equations 1 and 2 can be extended accordingly to

3 $$\Delta E_{ij}^{Z} = \frac{1}{2}\left( {Z_{ij} E_{ij}^{min} + Z_{ji} E_{ji}^{min} } \right) - \frac{1}{2}\left( {Z_{ii} E_{ii}^{min} + Z_{jj} E_{jj}^{min} } \right)$$

and

4 $$\Delta \left\langle {E_{ij} } \right\rangle^{Z} = \frac{1}{2}\left( {Z_{ij} \left\langle {E_{ij} } \right\rangle^{min} + Z_{ji} \left\langle {E_{ji} } \right\rangle^{min} } \right) - \frac{1}{2}\left( {Z_{ii} \left\langle {E_{ii} } \right\rangle^{min} + Z_{jj} \left\langle {E_{jj} } \right\rangle^{min} } \right)$$

respectively, where superscript *Z* denotes the coordination number extension in contrast to Eqs. 1 and 2, ${E}_{ij}^{min}={E}_{ji}^{min}$ and ${\langle {E}_{ij}\rangle }^{min}={\langle {E}_{ji}\rangle }^{min}$ but ${Z}_{ij}$ may be different from ${Z}_{ji}$, i.e. ${Z}_{ij}\ne {Z}_{ji}$.

Thus, the estimation of differential pair interaction energies requires two steps: (I) A calculation scheme to obtain the different (averaged) molecule pair interaction energies ${E}_{ij}^{min}$ (${\langle {E}_{ij}\rangle }^{min}$), and (II) a procedure to estimate the different coordination numbers ${Z}_{ij}$. In the following, the concrete implementation of a corresponding automated calculation pipeline for a selected molecular force field using the Tinker Molecular Modelling package is described. All dimer-related calculations are started with the global minimum energy conformers of the two constituent monomer molecules (see following “Global minimum energy monomers” section), where multiple dimer configurations are analyzed with these global minimum energy monomers (see following “Global minimum energy dimers” section) to obtain an approximate configuration for the global minimum energy dimer. The latter is achieved by optimizing this final approximate dimer configuration without constraints using all atomic degrees of freedom, so that the monomers are no longer confined to their individual global minimum energy conformers.

### Global minimum energy monomers

The global minimum energy conformers are derived in advance from conformer search procedures: STARTING from an initially defined chemically intuitive geometry of a monomer molecule, a first geometry improvement is achieved with Tinker *optimize* using the Optimally Conditioned Variable Metric (OCVM) optimization technique [by default with a root mean square (RMS) gradient of 0.01 kcal/mole/Å] [8]. The resulting locally optimized geometry is then transferred to a low-mode conformational search (LMOD) with Tinker *scan* to find the minimum energy conformer (where by default, the RMS gradient for the LMOD procedure is set to 0.0001 kcal/mole/Å, the energy threshold for local minima is set to 100 kcal/mole, torsion angles are automatically selected, and five eigenvectors are used for the local search) [9]. For small molecules with O(10) number of atoms, the detected minimum energy conformers usually coincide with the global minimum energy conformers.

### Global minimum energy dimers

**Fig. 1:**
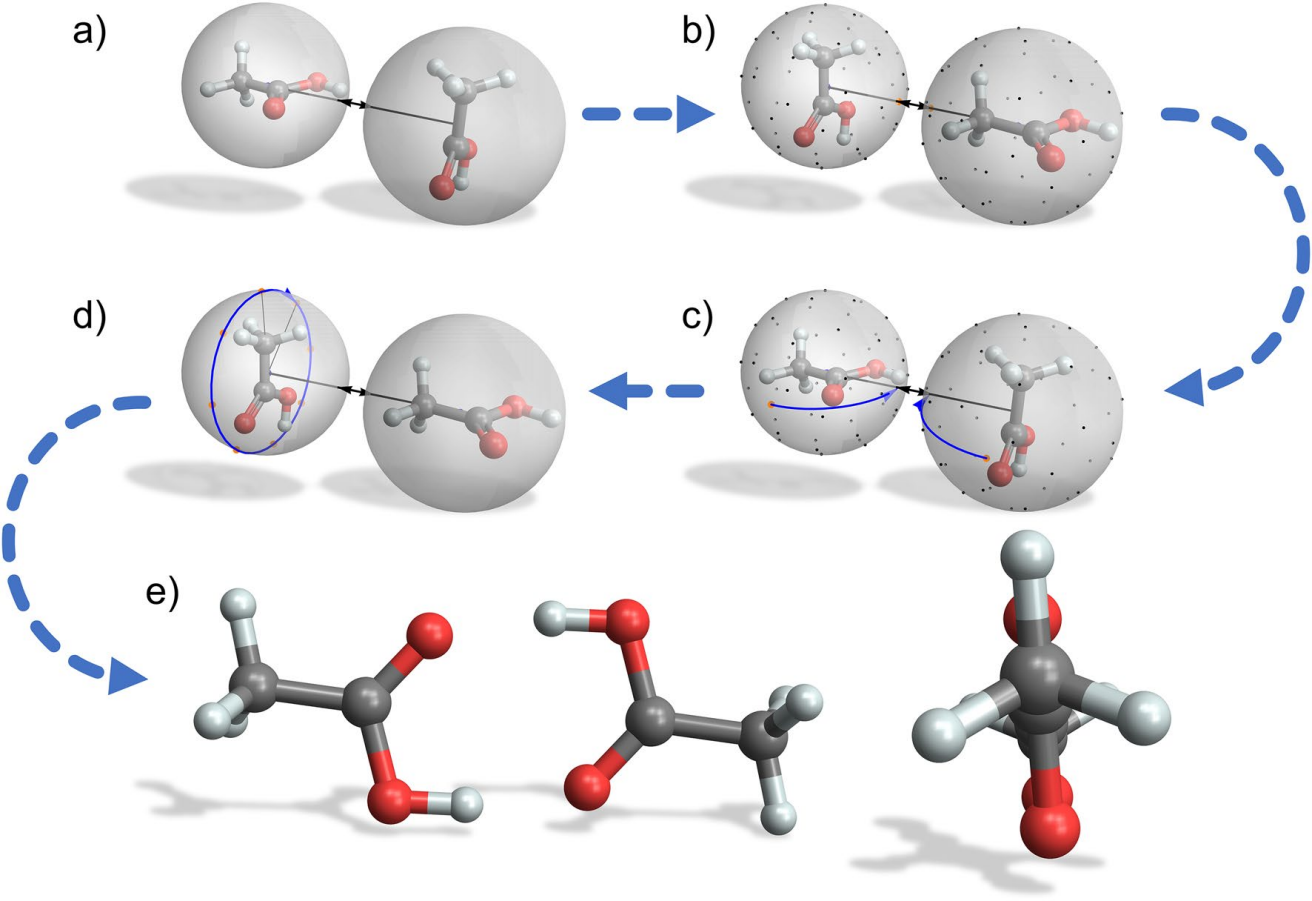
Configuration sampling for the acetic acid dimer: **a** An initial dimer of two acetic acid molecules is constructed with a distinct distance between the centers of both molecules (with each monomer being constrained to its individual global minimum energy conformer). **b** Spheres around the centers of the molecules are populated with a number ${N}_{sphere}$ of evenly distributed (equidistant) points on their surfaces. **c** The interaction energy is determined for every configuration where two adjacent spherical surface points and the centers of both molecules lie on a straight axis (which is achieved by corresponding rotations of the molecules around their centers). **d** For each single configuration in **c** the second molecule is also rotated for a number of angles ${N}_{rot}$ around the axis with a corresponding interaction energy calculation. **e** By varying and refining the distance between the molecule centers, the minimum energy dimer is determined and finally optimized to approximate the global minimum energy dimer without constraints using all atomic degrees of freedom. For the acetic acid dimer two perspectives of the finally optimized global minimum energy dimer with ${E}_{ij}^{min}$ are shown, using the MMFF94 force field [11]

**Fig. 2:**
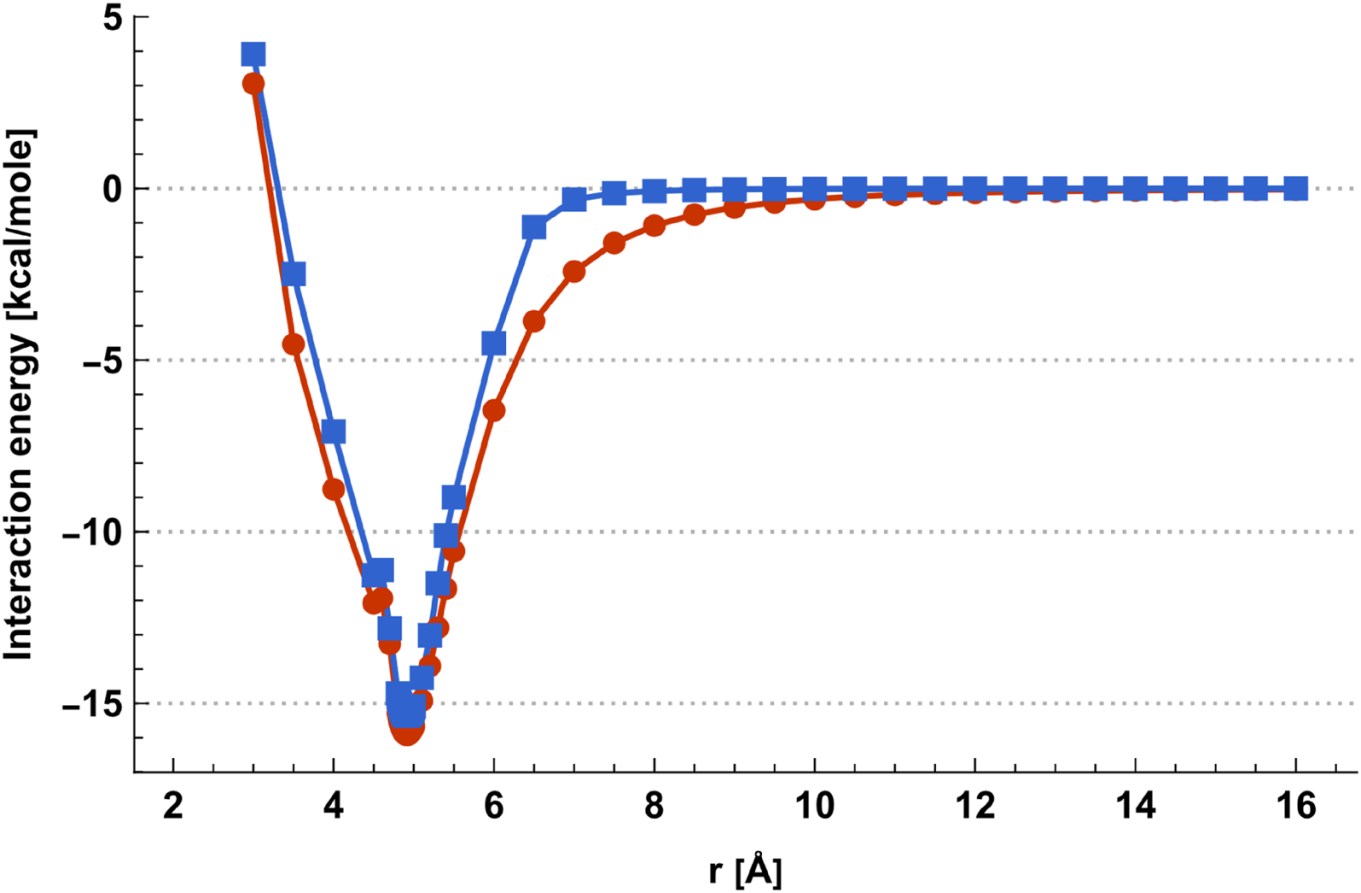
Acetic acid dimer. Red: ${E}_{ij}^{{C}^{*}}\left(r\right)$ for the minimum sampled energy configuration, Blue: Averaged interaction energy $\langle {E}_{ij}\rangle \left(r\right)$ for temperature $T=298 K$

To approximate the global minimum energy dimer of a pair of molecules $i$ and $j$, the centers of the molecules are positioned at different distances ranging (by default) from 3 to 16 Å in steps of 0.5 Å where the initial relative configuration of the two molecules is arbitrary. For each distance, a configuration sampling procedure is performed, which is sketched in Fig. 1. A (unit) sphere around each center is constructed with a number of ${N}_{sphere}$ evenly spaced points being generated on each sphere using a Fibonacci lattice as an adequate approximation (in comparison to a latitude–longitude lattice the surface points generated by a Fibonacci lattice are more evenly spaced with a smaller axial anisotropy) [10]. By rotating the molecules around their centers so that two adjacent spherical surface points and the centers of both molecules lie on a straight axis, ${N}_{sphere}\times {N}_{sphere}$ configurations are generated for which the corresponding interaction energies are determined by Tinker *analyze* (with settings to only compute the non-bonding interactions for a significantly accelerated performance). In addition, for each single configuration the second molecule is rotated for a number ${N}_{rot}$ of angles around the axis with corresponding interaction energy calculations, so that in total ${N}_{sphere}\times {N}_{sphere}\times {N}_{rot}$ spatial configurations are sampled for a single fixed distance between the monomer molecules (with each monomer being constrained to its individual global minimum energy conformer). The distance resolution is then refined around the distance with the detected minimum interaction energy dimer by reducing the step size from 0.5 to 0.1 Å, and then again from 0.1 to 0.01 Å (compare Fig. 2), so that a final near minimum energy dimer configuration is evaluated for the latter resolution. This resulting configuration ${C}^{*}$ is then optimized with Tinker *optimize* without constraints using all atomic degrees of freedom (i.e. the monomers are no longer confined to their individual global minimum energy conformers) to arrive at the force-field dependent approximation for ${E}_{ij}^{min}$ of the two molecules $i$ and $j$, the global minimum energy dimer using the specified force-field.

### Configuration sampling

If configuration sampling is desired for a specific distance ${r}_{fix}$ of the dimer molecules, the (already) obtained interaction energies of the ${N}_{sphere}\times {N}_{sphere}\times {N}_{rot}$ configurations for this distance may be averaged with Boltzmann weights ${w}_{C}\left({r}_{fix}\right)$ for a defined temperature (with ${\text{k}}_{\text{B}}$, the Boltzmann constant, and $\text{T}$, thermodynamic temperature) using ${E}_{ij}^{min}$:

5 $$w_{C} \left( {r_{fix} } \right) = {\text{e}}^{{ - \frac{{E_{ij}^{C} \left( {r_{fix} } \right) - E_{ij}^{min} }}{{{\text{k}}_{{\text{B}}} {\text{T}}}}}}$$

6 $$\left\langle {E_{ij} } \right\rangle \left( {r_{fix} } \right) = \frac{{\mathop \sum \nolimits_{C}^{{N_{sphere} \times N_{sphere} \times N_{rot} }} E_{ij}^{C} \left( {r_{fix} } \right) w_{C} \left( {r_{fix} } \right)}}{{\mathop \sum \nolimits_{C}^{{N_{sphere} \times N_{sphere} \times N_{rot} }} w_{C} \left( {r_{fix} } \right)}}$$

Then $\langle {E}_{ij}\rangle$ can be evaluated from the different $\langle {E}_{ij}\rangle \left(r\right)$. Figure 2 shows the quantitative results for the acetic acid dimer using the Merck molecular force field (MMFF94) [11].

### Coordination numbers

The coordination numbers ${Z}_{ij}$ are estimated by MD simulations. The simulation box construction is based on a pure water box at 298 K to get consistent results. A water molecule has a van der Waals volume of ${V}_{vdW}^{{H}_{2}O}=17.35$ Å^3^, in a pure water box it occupies a volume of ${V}_{box}^{{H}_{2}O}=30.00$ Å^3^ at 298 K due to its density [12] and molar mass [13]. This relation is mapped to other molecules $X$ with

7 $$\frac{{V_{box}^{{H_{2} O}} }}{{V_{vdW}^{{H_{2} O}} }} = \frac{{V_{box}^{X} }}{{V_{vdW}^{X} }}$$

so that the edge length $a$ of a cubic simulation box of $N$ molecules $X$ is given by

8 $$a = \sqrt[3]{{N \frac{{V_{box}^{{H_{2} O}} }}{{V_{vdW}^{{H_{2} O}} }} V_{vdW}^{X} }}$$

**Fig. 3:**
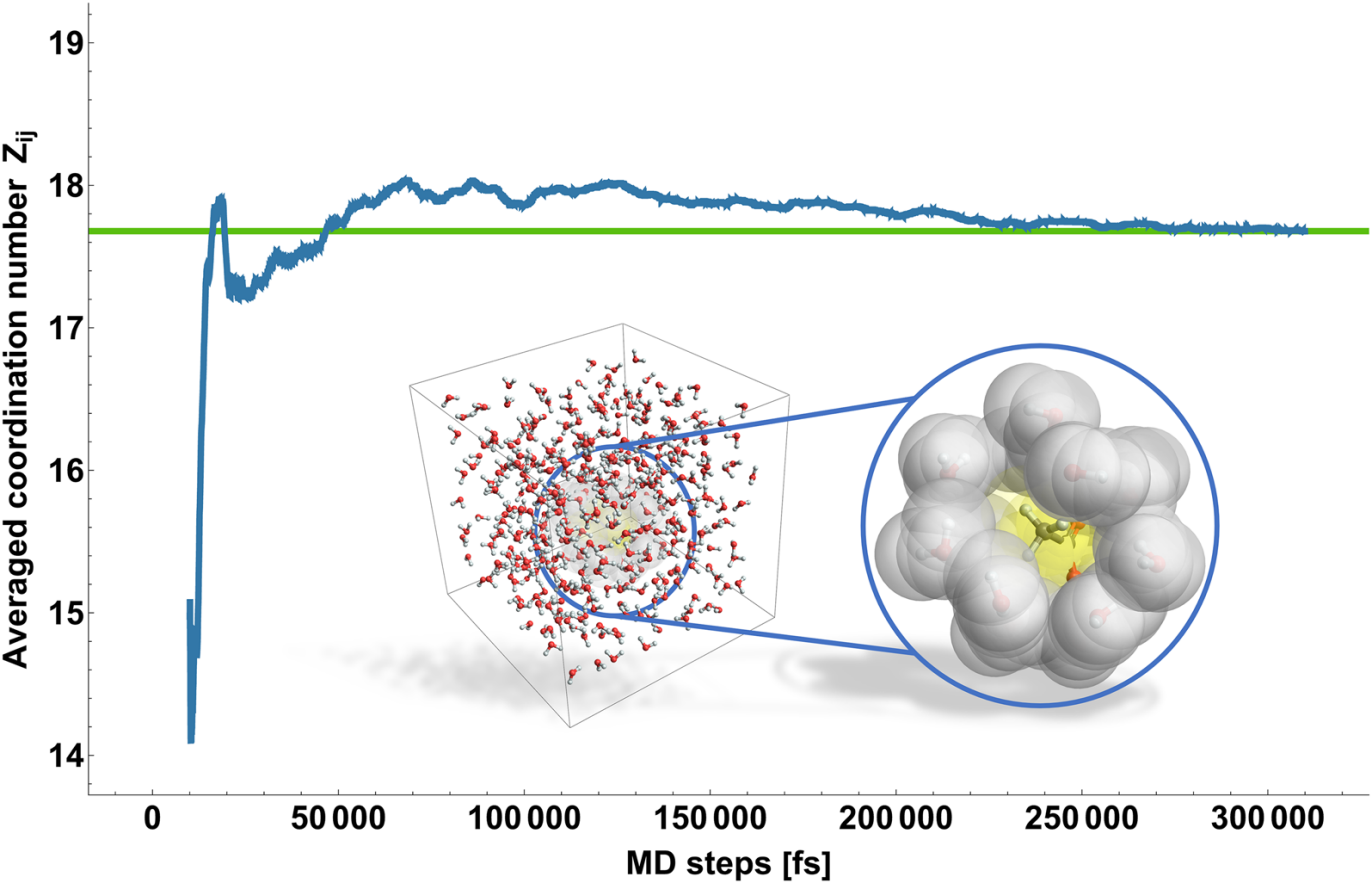
Averaged coordination number ${Z}_{ij}$ of a single acetic acid molecule in 400 water molecules starting after 10,000 initial steps (for box equilibration) with *a* “catch radius” of 1 Å using the MMFF94 force field. The averaged coordination number converges to a value of 17.7. Box graphics: Left: Snapshot of a simulation step of a single acetic acid molecule in 400 water molecules. Right: Magnification of the neighboring water molecules around the single acetic acid molecule. Yellow spheres: atoms of the single acetic acid molecule with their van der Waals radii. Grey spheres: Neighboring water molecules considered for the coordination number determination

The van der Waals volumes are approximated with the *VABCVolume* [14] descriptor of the Chemistry Development Kit (CDK) [15, 16]. A simulation box with a defined number $N$ of molecules $j$ (default is 400) and defined edge length $a$ is created using Tinker *xyzedit*. Then a single molecule $i$ is added to the box, where Tinker *xyzedit* automatically removes molecules $j$ to keep the defined density of the simulation box. The (possibly unfavorable) start configuration is optimized with Tinker *minimize* to avoid atomic contacts that could lead to instabilities. The following MD simulation uses Tinker *dynamics* with a step size of one femtosecond and an Andersen thermostat [17] for temperature equilibrium (default is 298 K). 10,000 (default) initial steps are used for box equilibration, followed by several hundred thousand steps with data recording (default is 400,000). For each recorded simulation step the number of molecules $j$ surrounding the single molecule $i$ is analyzed. This is done either by a “brute-force” counting approach or, alternatively, by the cell-index method with periodic boundary conditions [18]. The “brute-force” approach calculates the distances between each atom of the single molecule $i$ and each atom of all molecules $j$. Alternatively, the cell-index method only considers the (drastically reduced) distances between each atom of single molecule $i$ and the atoms of solvent molecules $j$ in neighbouring cells. The criterion for including a molecule $j$ as a relevant neighbor for the coordination number ${Z}_{ij}$ is based on the distance between its atoms and those of molecule $i$. If the distance of the respective atoms is less than or equal to the sum of their van der Waals radii plus an arbitrary “catch radius” (default is 1 Å), the molecules are considered neighbors. In Fig. 3, a snapshot of a simulation step is illustrated. For each simulation step, the number of neighboring molecules $j$ is determined. The average over all recorded steps is used to estimate the coordination number ${Z}_{ij}$, see Fig. 3.

### Flory–Huggins and mesoscopic repulsion parameters

Differential pair interaction energies may be directly utilized to estimate Flory–Huggins interaction parameters ${\chi }_{ij}$ by

9 $$\chi_{ij} = \frac{{\Delta \left\langle {E_{ij} } \right\rangle^{Z} }}{{{\text{k}}_{{\text{B}}} {\text{T}}}}$$

to describe polymer solutions [5], with $\Delta {\langle {E}_{ij}\rangle }^{Z}$ being defined in Eq. 4.

For “bridging the gap between atomistic and mesoscopic simulation” (Groot and Warren [6]), the interacting molecules can be identified with the particles of “bottom-up” mesoscopic Dissipative Particle Dynamics (DPD), where the microscopic Flory–Huggins interaction parameters ${\chi }_{ij}$ can be directly related to mesoscopic isotropic particle–particle repulsions ${a}_{ij}\left(T\right)$ (expressed in units of ${\text{k}}_{\text{B}}\text{T}$, with ${\rho }_{DPD}$ being the dimensionless DPD density, refer to [6] for details)

10 $$a_{ij} \left( T \right) = 75\frac{{{\text{k}}_{{\text{B}}} {\text{T}}}}{{\rho_{DPD} }} + 3.4965 {\text{k}}_{{\text{B}}} {\text{T}} \chi_{ij}$$

which determine the conservative DPD forces ${\underline{F}}_{ij}^{C,DPD}$:

11 $$\underline {F}_{ij}^{C} = \underline {F}_{ij}^{C,DPD} + \underline {F}_{ij}^{C,Bond}$$

12 $$\underline {F}_{ij}^{C,DPD} \left( {\underline {r}_{ij} } \right) = \left\{ {\begin{array}{*{20}l} {a_{ij} \left( {1 - r_{ij} } \right) \underline {r}_{ij}^{0} } & {{\text{for }}r_{ij} < 1} \\ 0 & {{\text{for }}r_{ij} \ge 1} \\ \end{array} } \right.$$

with ${\underline{F}}_{ij}^{C,DPD}$*,* ${\underline{F}}_{ij}^{C,Bond}$, soft repulsive DPD force and harmonic bond force on particle* i* exerted by particle* j*; ${a}_{ij}$, maximum isotropic repulsion between particles $i$ and $j$; ${\underline{r}}_{ij}={\underline{r}}_{i}-{\underline{r}}_{j}={r}_{ij }{\underline{r}}_{ij}^{0}$*;* ${\underline{r}}_{ij}^{0}$, unit vector. The numerical factor (3.4965) in Eq. 10 is derived from Eq. 24 in reference [6] where the inverse value (0.286) is given.

In interplay with the dissipative (frictional) forces ${\underline{F}}_{ij}^{D}$ and random forces ${\underline{F}}_{ij}^{R}$ the conservative forces ${\underline{F}}_{ij}^{C}$ guide the trajectories ${\underline{r}}_{i}\left(t\right)$ of the DPD particles according to Newton’s equation of motion:

13 $$m_{i} \frac{{d^{2} \underline {r}_{i} }}{{dt^{2} }} = \underline {F}_{i} = \mathop \sum \limits_{j = 1,j \ne i}^{N} \left( {\underline {F}_{ij}^{C} + \underline {F}_{ij}^{D} + \underline {F}_{ij}^{R} } \right)$$

with ${m}_{i}$*,* ${\underline{r}}_{i}$, mass and position vector of particle $i$; $t$, time; ${\underline{F}}_{i}$, total force on particle $i$ exerted by other particles $j$; $N$, total number of particles in simulation; ${\underline{F}}_{ij}^{C}$*, *${\underline{F}}_{ij}^{D}$*,* ${\underline{F}}_{ij}^{R}$, conservative, dissipative, and random force on particle $i$ exerted by particle $j$*.*

Thus, the described calculation pipeline may be applied to construct a force-field-based particle set for DPD simulations.

### Pipeline code availability

The pipeline code is written in Java and openly available at [19]. A dedicated installer executable for the Java pipeline code, which comprises a full Java runtime environment, is available at [20] for the Windows operating system. For Linux operating systems a zip file is available at [20]. According to its licensing terms the Tinker executables for *optimize*, *scan*, *analyse* etc. have to be downloaded from its website [21] into a specified pipeline directory: a detailed instruction how to perform this is provided at [22]. A set of stand-alone Mathematica notebooks [23] for result visualizations is provided at [24]. A test pipeline is available at [19] to ensure proper installation.

### Pipeline calculation performance

Calculation of a full single differential pair interaction energy for the force fields MM3, MMFF94 and OPLS-AA with default settings (${N}_{sphere}\times {N}_{sphere}\times {N}_{rot}=144\times 144\times 16=\text{331,776}$ dimer configurations for each fixed distance to approximate the intermolecular interaction energies, 10,000 equilibration steps and 400,000 simulation steps for the MD simulations to estimate the coordination number with 400 molecules in the box) takes several hours, where the AMOEBA09 force field requires a multiple. Since the pipeline supports comprehensive calculation parallelization for a set of monomer molecules, a single differential pair interaction energy can be obtained on average in less than an hour on a 64-core AMD Ryzen™ Threadripper™ PRO 5995 CPU workstation [25].

### DFT calculations for result evaluation

DFT calculations are performed with Gaussian 16 [26] and analyzed with GaussView 6 [27]. All molecular geometries are optimized using the dispersion-corrected wB97XD functional [28] with the 6–311++G(d,p) basis set where counterpoise calculations are used to obtain basis set superposition error (BSSE) corrected interaction energies. All Gaussian jobs files used are openly available at [29].

### DPD simulations for result evaluation

All DPD simulations of this study are performed with the MFsim simulation system [30] using the Jdpd simulation kernel [31]. All constructed particle sets and MFsim simulation jobs are openly available at [32].

## Results and discussion

To demonstrate the applicability of the different steps of the calculation pipeline, several small molecules are selected: Water (abbreviated H2O), methane (Me), ethane (Et), methanol (MeOH), dimethyl ether (Me2O) and the acetic acid dimer (HAc). The calculation results for this molecule set are evaluated and compared with alternative approaches and experimental results. All averaged energies and MD simulations refer to a temperature of $T=298 K$. All intermolecular energy calculations were performed with ${N}_{sphere}\times {N}_{sphere}\times {N}_{rot}=144\times 144\times 16=\text{331,776}$ dimer configurations for each fixed distance of the molecule centers.

### Acetic acid dimer

**Fig. 4:**
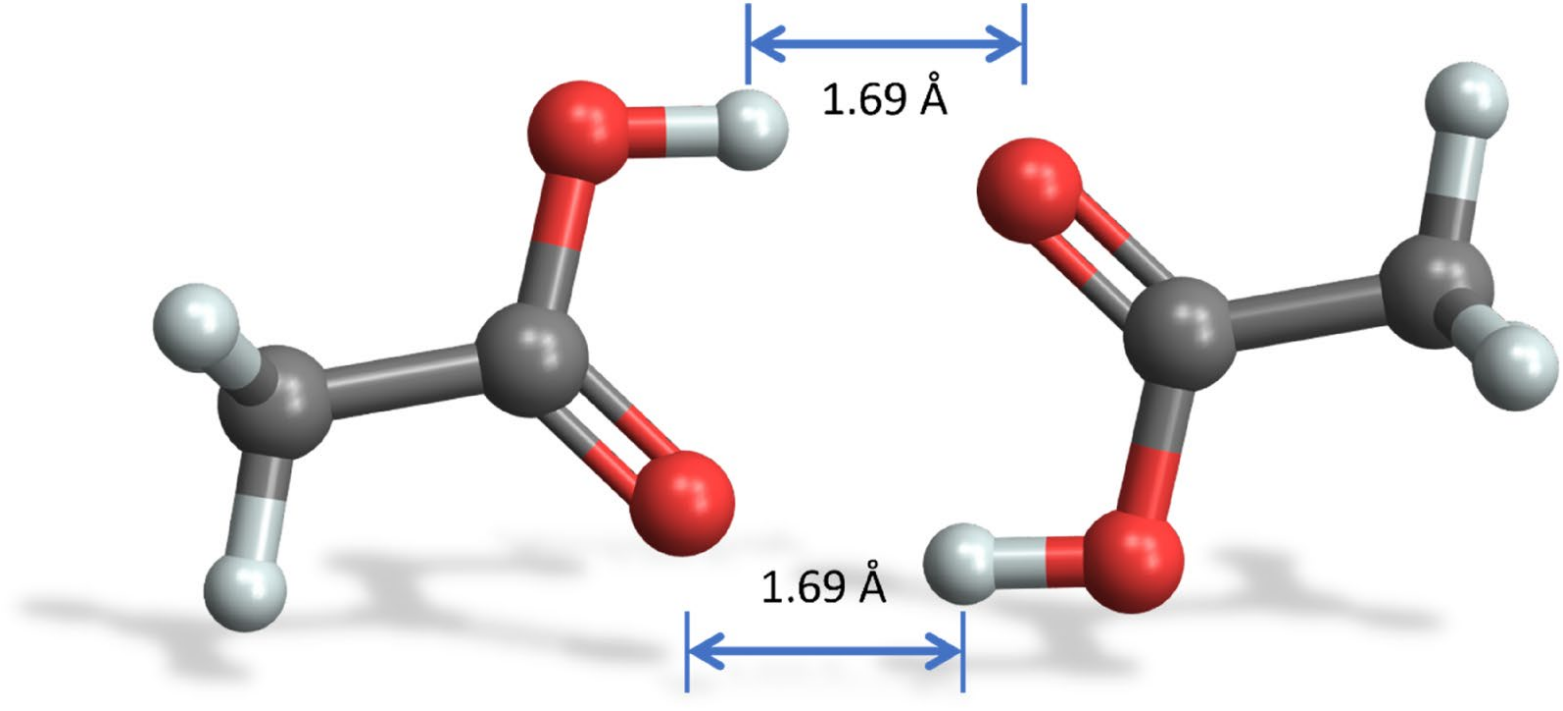
Minimal energy configuration ${C}^{*}$ of the acetic acid dimer with two hydrogen bonds

The acetic acid dimer is stabilized by two hydrogen bonds and has a planar geometry. Calculation results with the MMFF94 force field are shown in Figs. 2 and 4. Figure 2 depicts the minimal energy configuration ${C}^{*}$ energies ${E}_{ij}^{{C}^{*}}\left({r}_{fix}\right)$ for each specific distance ${r}_{fix}$ in red, exhibiting a single minimum at *r*_*fix*_ = 4.91 Å with $E_{ij}^{C*}$ (4.91 Å) = − 15.9 kcal/mole*.* The corresponding averaged intermolecular energies $\langle {E}_{ij}\rangle \left({r}_{fix}\right)$ are shown in blue, where the single minimum distance coincides at *r*_*fix*_ = 4.91 Å with $\left\langle {E_{ij} } \right\rangle$ (4.91 Å) = − 15.4 kcal/mole in this case. The two hydrogen bonds of the minimal energy configuration ${C}^{*}$ have a length of 1.69 Å and a distance of 2.63 Å between the corresponding donor and acceptor oxygen atoms (see Fig. 4), which is close to the experimental values of 2.68 Å [33].

**Fig. 5:**
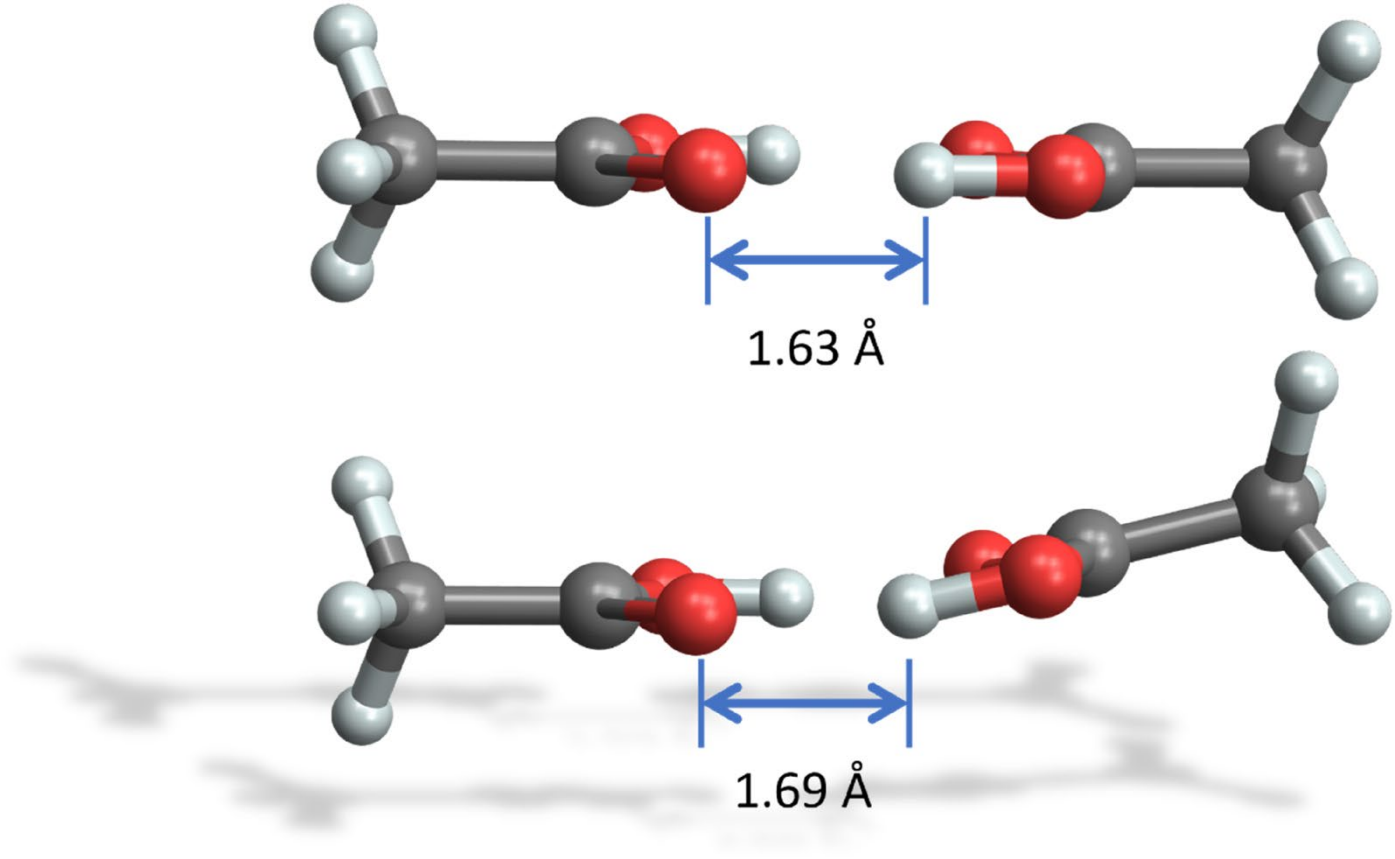
Acetic acid dimer with the MMFF94 force field. Bottom: minimal sampled energy configuration ${C}^{*}$ with ${E}_{ij}^{{C}^{*}}$ (4.91 Å) = − 15.9 kcal/mole. Top: optimized global minimum energy configuration ${C}_{min}$ with ${E}_{ij}^{min}={E}_{ij}^{{C}_{min}}$ (4.91 Å) = − 17.6 kcal/mole

With the final optimization of the near minimal energy configuration ${C}^{*}$ the global MMFF94 force-field-based minimum energy configuration ${C}_{min}$ with ${E}_{ij}^{min}={E}_{ij}^{{C}_{min}}\left({r}_{min}\right)=-17.6$ kcal/mole is obtained, which is 1.7 kcal/mole below the sampled minimal energy ${C}^{*}$ configuration: the finally optimized dimer keeps the distance of *r*_*min*_ = 4.91 Å but shows a planar geometry with a slightly reduced hydrogen bond length of 1.63 Å and a distance of 2.62 Å between the donor and acceptor oxygen atoms (see Fig. 5).

### Mutual dimer calculations

Table 1 presents the mutual dimer calculations: it comprises $\langle {E}_{ij}\rangle$ and ${E}_{ij}^{{C}^{*}}$ for the detected minima (compare Fig. 2) as well as the global force-field-based energy minimum ${E}_{ij}^{min}$ for force fields MMFF94, AMOEBA09, MM3 [34] and OPLS-AA [35, 36] with the water models TIP3P [37] and TIP5P [38]. As expected, the nonpolar pure alkyl (methane and ethane) dimers exhibit only small interaction energies, the acetic acid dimer with two hydrogen bonds shows the largest interaction, and the polar dimers are in between. There are clear differences between the force fields, with the OPLS-AA (TIP5P) interactions for the alcohol–water dimers being the strongest. On average, MM3 differs most significantly from the other force fields.

**Table 1:** Force-field based intermolecular interaction energies in kcal/mole for the different dimers (averages are obtained at $T=298 K$)

Dimer	MMFF94			MM3			AMOEBA09			OPLS-AA (TIP3P)			OPLS-AA (TIP5P)		
	$\langle {E}_{ij}\rangle$	${E}_{ij}^{{C}^{*}}$	${E}_{ij}^{min}$	$\langle {E}_{ij}\rangle$	${E}_{ij}^{{C}^{*}}$	${E}_{ij}^{min}$	$\langle {E}_{ij}\rangle$	${E}_{ij}^{{C}^{*}}$	${E}_{ij}^{min}$	$\langle {E}_{ij}\rangle$	${E}_{ij}^{{C}^{*}}$	${E}_{ij}^{min}$	$\langle {E}_{ij}\rangle$	${E}_{ij}^{{C}^{*}}$	${E}_{ij}^{min}$
Et–Et	− 0.4	− 0.8	− 0.8	− 0.4	− 0.8	− 0.8	− 0.7	− 1.3	− 1.4	− 0.6	− 1.2	− 1.2			
EtOH–Et	− 0.4	− 1.0	− 1.0	− 0.4	− 0.9	− 0.9	− 0.9	− 2.0	− 2.1	− 0.7	− 1.5	− 1.5			
EtOH–EtOH	− 4.3	− 6.2	− 6.5	− 5.8	− 7.3	− 7.5	− 4.0	− 6.2	− 6.6	− 4.8	− 6.8	− 7.3			
H2O–Et	− 0.2	− 0.5	− 0.5	− 0.2	− 0.5	− 0.5	− 0.5	− 1.3	− 1.3	− 0.3	− 0.9	− 0.9	− 0.3	− 0.8	− 0.8
H2O–EtOH	− 4.9	− 6.3	− 6.7	− 6.3	− 7.5	− 7.7	− 4.8	− 6.4	− 6.5	− 5.4	− 6.7	− 7.3	− 5.5	− 7.6	− 10.9
H2O–H2O	− 5.0	− 6.3	− 6.8	− 5.9	− 7.2	− 7.4	− 3.3	− 4.9	− 5.0	− 5.1	− 6.5	− 6.9	− 4.8	− 6.5	− 7.3
Me–Et	− 0.3	− 0.6	− 0.6	− 0.3	− 0.6	− 0.6	− 0.4	− 0.9	− 0.9	− 0.4	− 0.8	− 0.8			
Me–EtOH	− 0.3	− 0.7	− 0.7	− 0.3	− 0.7	− 0.7	− 0.6	− 1.7	− 1.7	− 0.5	− 1.0	− 1.0			
Me–H2O	− 0.2	− 0.3	− 0.3	− 0.2	− 0.3	− 0.3	− 0.4	− 1.2	− 1.2	− 0.2	− 0.6	− 0.6	− 0.2	− 0.5	− 0.5
Me–Me	− 0.2	− 0.4	− 0.4	− 0.2	− 0.4	− 0.4	− 0.4	− 0.5	− 0.5	− 0.3	− 0.5	− 0.5			
Me2O–Et	− 0.4	− 1.0	− 1.0	− 0.4	− 0.9	− 1.0	− 0.9	− 1.9	− 2.0	− 0.8	− 1.4	− 1.4			
Me2O–EtOH	− 3.8	− 5.6	− 5.9	− 5.4	− 6.6	− 6.7	− 3.3	− 5.6	− 5.9	− 3.1	− 5.2	− 5.6			
Me2O–H2O	− 4.5	− 5.8	− 6.1	− 5.0	− 6.2	− 6.3	− 4.3	− 6.0	− 6.2	− 3.9	− 5.2	− 5.4	− 3.2	− 4.4	− 4.5
Me2O–Me	− 0.3	− 0.7	− 0.7	− 0.3	− 0.7	− 0.7	− 0.6	− 1.5	− 1.6	− 0.5	− 1.0	− 1.0			
Me2O–Me2O	− 1.2	− 2.4	− 2.4	− 0.7	− 1.5	− 1.6	− 1.6	− 3.1	− 3.2	− 1.5	− 2.4	− 2.4			
MeOH–Et	− 0.3	− 0.8	− 0.8	− 0.3	− 0.7	− 0.7	− 0.7	− 1.7	− 1.8	− 0.6	− 1.4	− 1.4			
MeOH–EtOH	− 4.7	− 6.2	− 6.4	− 5.9	− 7.3	− 7.5	− 4.6	− 6.4	− 6.7	− 5.1	− 6.7	− 7.2			
MeOH–H2O	− 4.6	− 6.0	− 6.5	− 6.0	− 7.6	− 7.6	− 3.8	− 5.6	− 5.8	− 4.9	− 6.4	− 6.9	− 6.4	− 8.0	− 10.8
MeOH–Me	− 0.3	− 0.6	− 0.6	− 0.3	− 0.5	− 0.5	− 0.5	− 1.4	− 1.4	− 0.4	− 0.9	− 0.9			
MeOH–Me2O	− 4.2	− 5.7	− 5.9	− 5.4	− 6.6	− 6.7	− 4.0	− 5.8	− 6.2	− 3.7	− 5.2	− 5.5			
MeOH–MeOH	− 4.3	− 5.9	− 6.1	− 5.8	− 7.1	− 7.2	− 3.7	− 5.6	− 5.8	− 4.5	− 6.2	− 6.6			
HAc–HAc	− 15.4	− 15.9	− 17.6	− 16.9	− 17.4	− 18.1	− 15.4	− 15.8	− 18.0	− 11.4	− 12.2	− 13.6			

**Fig. 6:**
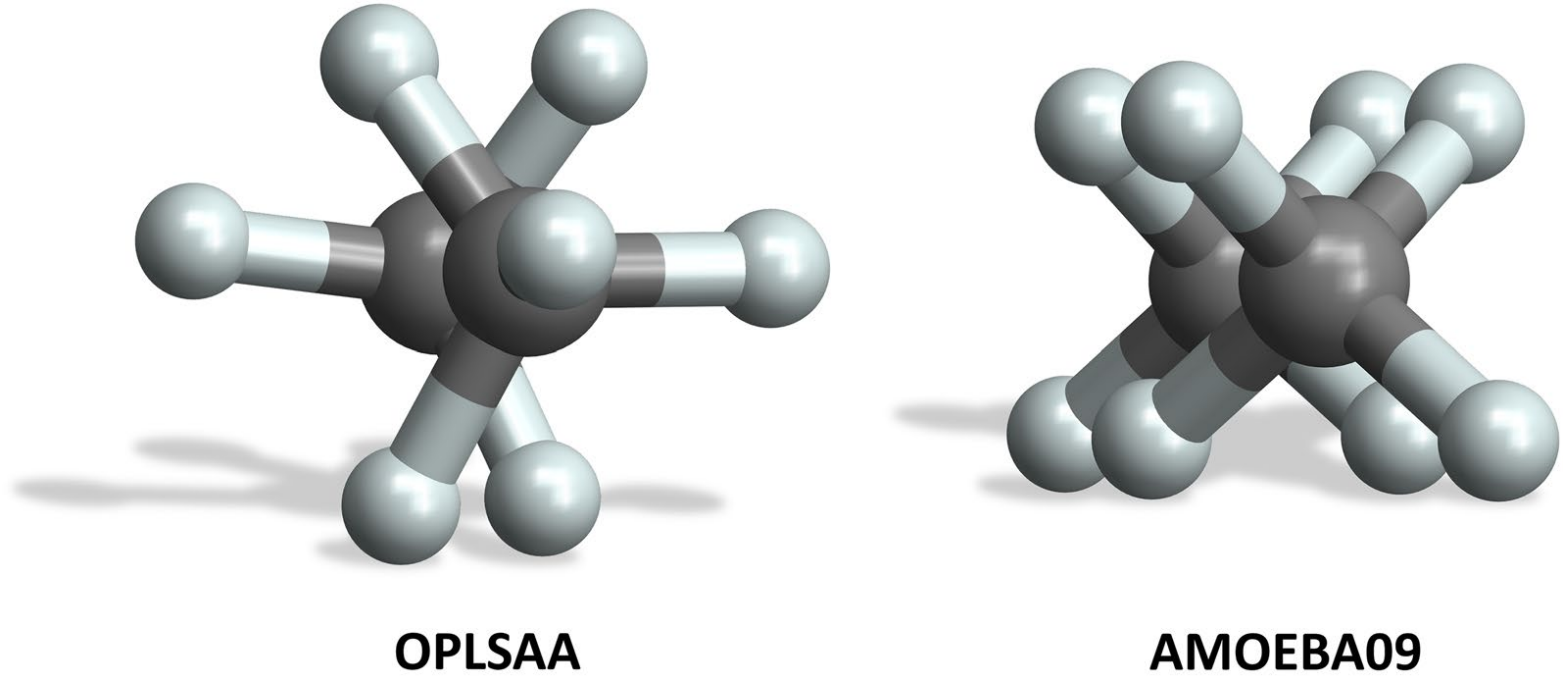
Minimum energy ${E}_{ij}^{{C}^{*}}$ configuration for the Me–Me dimer: Left: OPLS-AA force field with staggered configuration. Right: AMOEBA09 force field with eclipsed configuration

For the HAc–HAc and the Me–Me dimer the results for force fields OPLS-AA, AMOEBA09 and MMFF94 were compared with corresponding DFT single point calculations (denoted DFT sp). In addition, the spatial configurations with ${E}_{ij}^{min}$ were used as start geometries for DFT geometry optimizations (denoted DFT opt), see Table 2. The DFT calculations indicate that the automated pipeline leads to acceptable near minimum energies and corresponding spatial configurations—with individual exceptions: in contrast to the MMFF94 and OPLS-AA force fields, AMOEBA09 results in an eclipsed minimum energy configuration for the Me–Me dimer instead of a staggered one (see Fig. 6, this eclipsed configuration is maintained by the DFT geometry optimization), but this finding has no significant influence on the subsequent investigations due to its low energetic effect (see Table 2). The difference in interaction energies between the DFT opt and the OPLS-AA force field result for the HAc–HAc dimer is most significant.

**Table 2:** Force-field based interaction energies ${E}_{ij}^{{C}^{*}}$ *and* ${E}_{ij}^{min}$ with corresponding DFT interaction energies and configuration measures

	Dimer	${E}_{ij}^{{C}^{*}} \left[kcal/mole\right]$		Distance [Å]
		DFT sp	Force field	DFT = force field = fixed
OPLS-AA	Me–Me	− 0.6	− 0.5	3.57^b^
	HAc–HAc	− 14.5	− 12.2	2.69^a^
AMOEBA09	Me–Me	− 0.5	− 0.5	3.74^b, c^
	HAc–HAc	− 15.8	− 15.8	2.76^a^
MMFF94	Me–Me	− 0.6	− 0.4	3.74^b^
	HAc–HAc	− 11.5	− 15.9	2.63^a^

	Dimer	${E}_{ij}^{min} \left[kcal/mole\right]$		Distance [Å]
		DFT sp	Force field	DFT = force field = fixed
OPLS-AA	Me–Me	− 0.6	− 0.5	3.57^b^
	HAc–HAc	− 15.7	− 13.6	2.68^a^
AMOEBA09	Me–Me	− 0.5	− 0.5	3.74^b, c^
	HAc–HAc	− 17.6	− 18.0	2.73^a^
MMFF94	Me–Me	− 0.6	− 0.4	3.73^b^
	HAc–HAc	− 15.1	− 17.6	2.62^a^

	Dimer	DFT opt	Force field	DFT	Force field	Exp
OPLS-AA	Me–Me	− 0.6	− 0.5	3.57^b^	3.57^b^	3.85^b^
	HAc–HAc	− 19.8	− 13.6	2.68^a^	2.68^a^	2.68^a^
AMOEBA09	Me–Me	− 0.5	− 0.5	3.82^b,c^	3.74^b,c^	3.85^b^
	HAc–HAc	− 19.8	− 18.0	2.68^a^	2.73^a^	2.68^a^
MMFF94	Me–Me	− 0.6	− 0.4	3.57^b^	3.73^b^	3.85^b^
	HAc–HAc	− 19.8	− 17.6	2.68^a^	2.62^a^	2.68^a^

a: Hydrogen bond length (distance oxygen to oxygen), b: Distance between carbon atoms, c: Eclipsed minimum energy configuration

The Me-Me dimer distance denotes the distance between the carbon atoms (b), while the HAc-HAc dimer distance refers to the specific hydrogen bond length (labelled a). Experimental values (denoted Exp.) are taken from [29] for acetic acid and [33, 39] for methane. For the Me–Me dimer the AMOEBA09 force field yields an eclipsed minimum energy configuration instead of a staggered one (labelled c)

### Coordination numbers ${Z}_{ij}$

Table 3 contains the mutual coordination numbers ${Z}_{ij}$ (at $T=298 K$), where a single molecule *i* is surrounded by 400 molecules *j*, using the MMFF94, OPLS-AA (with TIP3P and TIP5P water models), and AMOEBA09 force fields. For comparison, static packing [40] results are included which were taken from previous research [41] and obtained with the commercial Blends software of Materials Studio [42] using the Condensed-phase Optimized Molecular Potentials for Atomistic Simulation Studies (COMPASS) force field [43].

**Table 3:** Coordination numbers ${Z}_{ij}$ (at $T=298 K$)

Dimer	${Z}_{ij}$					${Z}_{ji}$				
	MMFF94	OPLS-AA (TIP3P)	OPLS-AA (TIP5P)	AMOEBA 09	STATIC PACKING	MMFF94	OPLS-AA (TIP3P)	OPLS-AA (TIP5P)	AMOEBA 09	STATIC PACKING
Et–Et	12.0	11.8	11.8	12.0	5.6	12.0	11.8	11.8	12.0	5.6
EtOH–Et	12.9	12.6	12.6	12.6	5.8	10.4	10.3	10.3	10.2	5.3
EtOH–EtOH	11.4	11.1	11.1	11.0	5.6	11.4	11.1	11.1	11.0	5.6
H2O–Et	8.7	8.2	8.2	8.4	4.5	16.3	16.4	17.3	17.2	6.9
H2O–EtOH	7.2	7.0	7.0	6.8	4.3	17.9	17.8	18.4	18.7	7.3
H2O–H2O	11.0	10.9	10.8	10.8	5.5	11.0	10.9	10.8	10.8	5.5
Me–Et	10.0	9.8	9.8	9.7	4.9	14.9	14.8	14.8	14.6	6.3
Me–EtOH	8.8	8.6	8.6	8.3	4.7	16.1	15.8	15.8	15.6	6.6
Me–H2O	13.3	13.2	14.2	13.7	6.1	10.6	10.2	10.2	10.1	5.0
Me–Me	12.3	12.1	12.1	11.9	5.6	12.3	12.1	12.1	11.9	5.6
Me2O–Et	13.0	12.8	12.8	12.7	5.7	11.0	10.7	10.7	10.7	5.3
Me2O–EtOH	11.5	11.1	11.1	11.0	5.5	11.5	11.4	11.4	11.4	5.6
Me2O–H2O	18.0	17.8	18.7	18.5	7.3	7.5	7.2	7.3	7.5	4.3
Me2O–Me	16.3	15.9	15.9	15.7	6.6	9.3	8.9	8.9	8.8	4.7
Me2O–Me2O	12.2	11.6	11.6	11.7	5.6	12.2	11.6	11.6	11.7	5.6
MeOH–Et	11.0	10.8	10.8	10.8	5.2	12.9	12.7	12.7	12.3	5.9
MeOH–EtOH	9.4	9.3	9.3	9.3	5.0	13.3	13.4	13.4	13.0	6.2
MeOH–H2O	14.8	14.7	15.1	15.2	6.5	8.2	8.1	8.0	8.4	4.7
MeOH–Me	13.6	13.4	13.4	13.2	6.0	10.5	10.5	10.5	9.9	5.2
MeOH–Me2O	10.0	9.7	9.7	9.8	5.0	13.8	13.6	13.6	13.4	6.1
MeOH–MeOH	11.5	11.2	11.2	11.3	5.6	11.5	11.2	11.2	11.3	5.6
HAc–HAc	9.7	10.2	10.2	10.1	5.6	9.7	10.2	10.2	10.1	5.6

The static packing results from previous research are added for comparison only

**Fig. 7:**
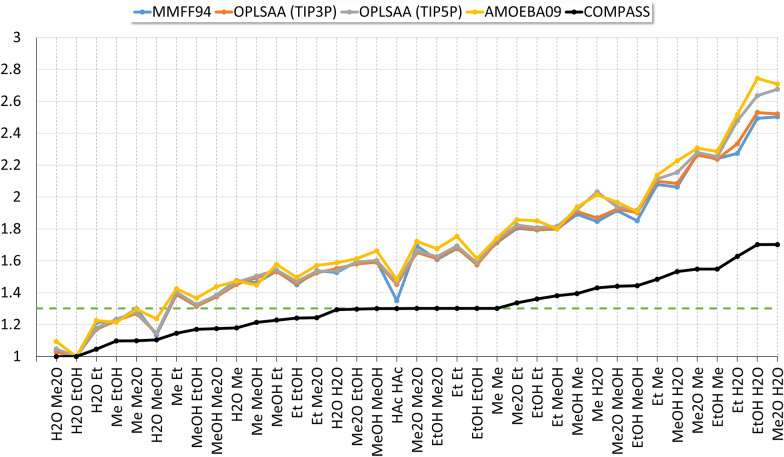
Coordination numbers ${Z}_{ij}$, scaled to 1 for each force field. The black line indicates the COMPASS static packing coordination numbers for the homo dimers

**Fig. 8:**
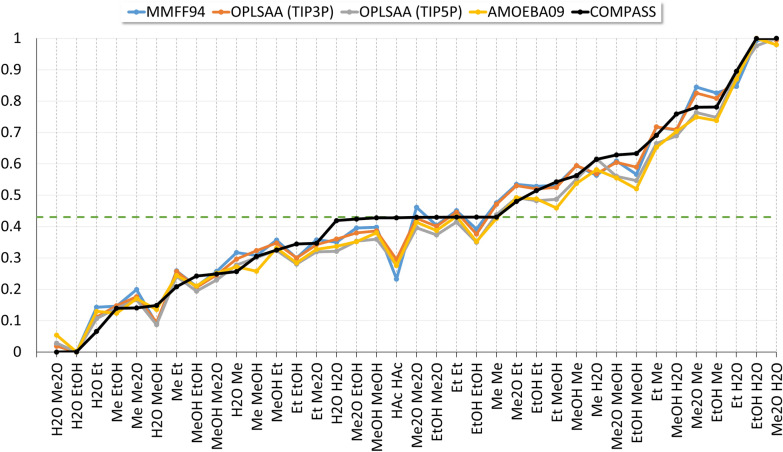
Coordination numbers ${Z}_{ij}$, linearly mapped to interval [0,1] for each force field. The black line indicates the COMPASS static packing coordination numbers for the homo dimers

Figures 7 and 8 show the similarity of trends in coordination number assignment. While the MD-derived coordination numbers are similar for the different force fields used, the results for the COMPASS static packing approach are significantly reduced by about 50%. The spread of the values derived from MD (Fig. 7) is significantly higher than that of the static packing approach. Interestingly, linear mapping of the coordination numbers to the interval [0,1] yields an approximate overlap of the results (Fig. 8).

### Repulsion parameters ${a}_{ij}$

For the different force fields, particle sets for mesoscopic DPD simulations with the isotropic mutual repulsions ${a}_{ij}$ for methane (Me), ethane (Et), methanol (MeOH), ethanol (EtOH), dimethyl ether (Me2O), and water (H2O) were constructed. The off-diagonal ${a}_{ij}$ values of a particle set were linearly scaled with MFsim [30] so that the maximum absolute deviation between the smallest ${a}_{ij}$ value and the diagonal values ${a}_{ii}=24.83$ (for a thermodynamic temperature of 298 K) is 20. For the OPLS-AA force field the water models TIP3P and TIP5P were considered, denoted OPLS-AA (TIP3P) and OPLS-AA (TIP5P). An additional OPLS-AA (TIP5P) particle set, denoted OPLS-AA (TIP5P ${Z}_{ij}=1$), was created which is solely based on the minimal averaged intermolecular energy $\langle {E}_{ij}\rangle$ with a fixed coordination number ${Z}_{ij}=1$ for all dimers. With force field MM3, interaction energies can be calculated only, so a combined particle set was created using MM3 for interaction energy calculation and the MMFF94 force field for MD-based coordination number estimation, denoted MM3/MMFF94. The particle set from [41, 44], based on the COMPASS force field, is used for comparison.

**Fig. 9:**
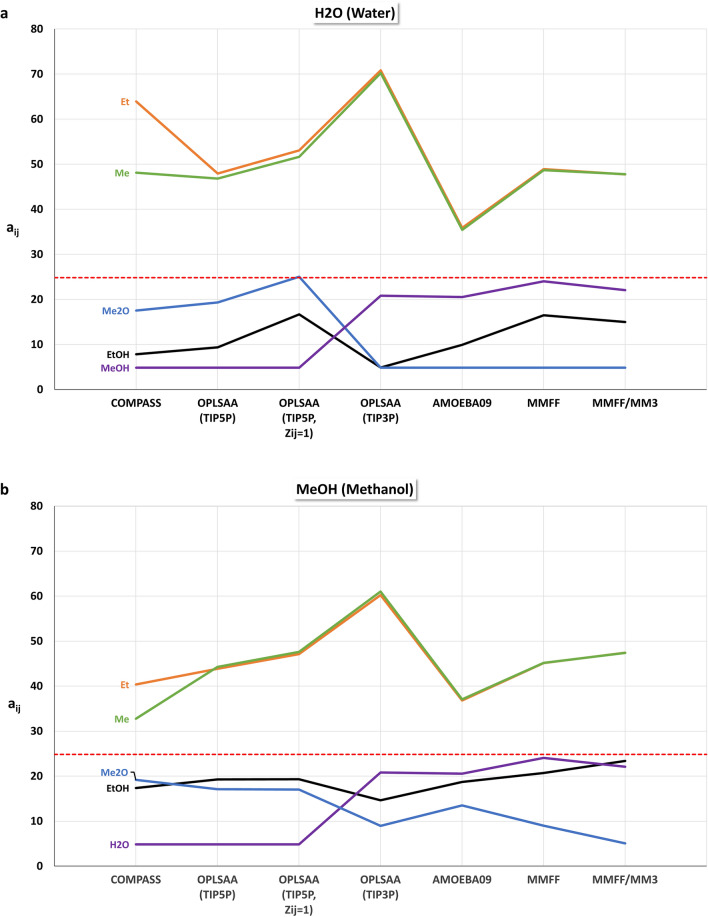

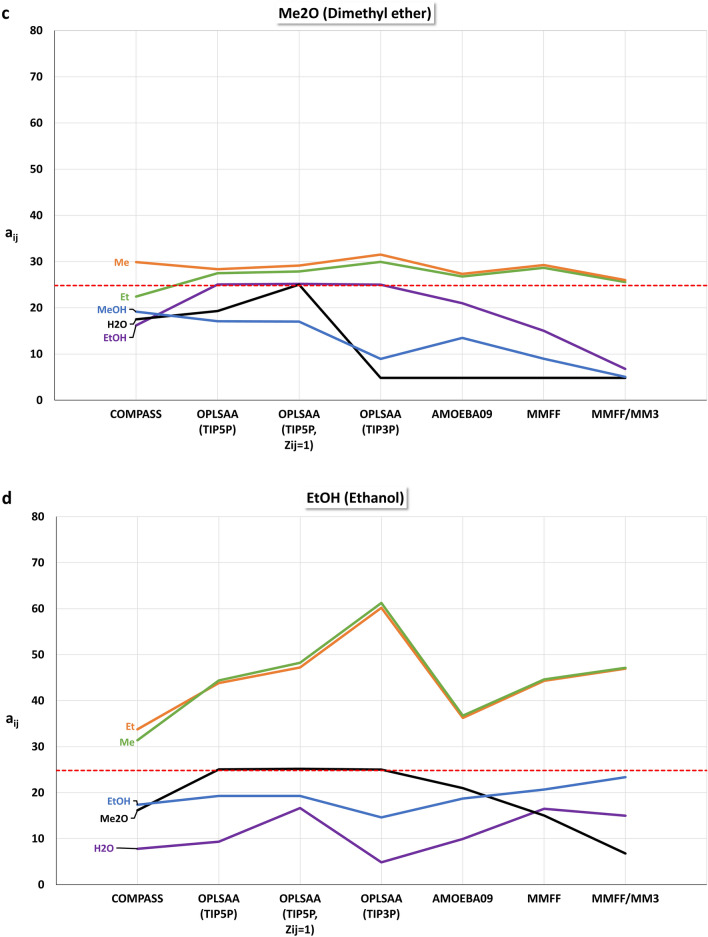

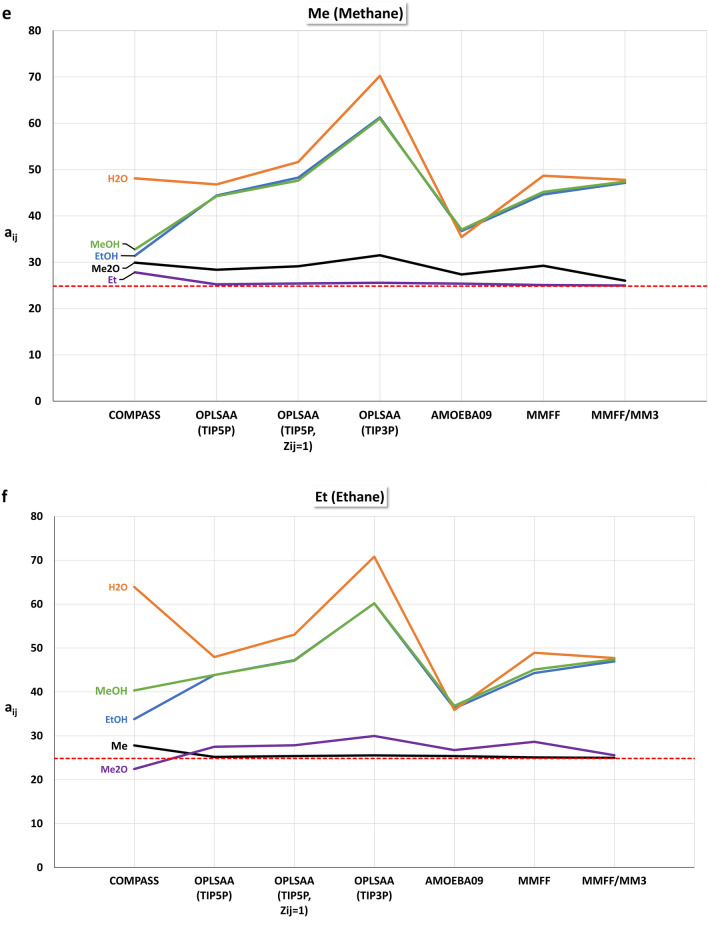
Scaled repulsion parameters ${a}_{ij}$. The red dashed line indicates the diagonal value of 24.83 for homo dimers

Figure 9a–f display the different repulsion parameters ${a}_{ij}$. The red dashed line indicates the diagonal value of 24.83. A crucial difference between the different particle sets is the water–methanol (H2O–MeOH) repulsion: for the COMPASS, OPLS-AA (TIP5P) and OPLS-AA (TIP5P, ${Z}_{ij}=1$) force fields, this repulsion is the smallest one (and thus the base value for off-diagonal repulsion parameter scaling), whereas for force fields OPLS-AA (TIP3P), AMOEBA09, MMFF94, and MM3/MMFF94 the water–dimethyl ether (H2O–Me2O) repulsion is minimal. Interestingly, the different water models TIP3P and TIP5P of the OPLS-AA force field exhibit this crucial difference, emphasizing their relevance. Note, that the differences of the non-water repulsions for OPLS-AA (TIP3P) and OPLS-AA (TIP5P) are caused by the different scaling due to these different base values for off-diagonal repulsion parameter scaling. The COMPASS force field exhibits an extraordinary difference for the water–methane (H2O–Me) and water–ethane (H2O–Et) repulsions, which is otherwise not visible. A significant difference between the OPLS-AA (TIP5P) and OPLS-AA (TIP5P, ${Z}_{ij}=1$) force field is the water–dimethyl ether (H2O–Me2O) repulsion. These obvious differences between the force fields lead to different levels of usefulness for DPD simulation approaches, where even a single difference in dimer interaction can become a decisive factor.

### DPD simulations

The created particle sets were used for DPD simulations of mixtures of water with the non-ionic polyoxyethylene alkyl ether surfactant C_10_E_4_, where “C_10_” indicates the number of 10 carbon atoms in the alkyl chain of the lyophobic part, and “E_4_” represents the number of 4 lyophilic ethylene oxide units [44]. A stable lamellar L_α_ phase is formed by a C_10_E_4_/water mixture around 298 K with a C_10_E_4_ mass fraction of 0.75. The performance of the particle sets for the different force fields may be evaluated by monitoring the emergent formation of C_10_E_4_ bilayers from initial random mixing in the simulation box [44]. The DPD simulations are carried out with the settings outlined in [44], using the SPICES 9Me–4Me2O–MeOH fragmentation for C_10_E_4_ [45, 46], an integration step size of 0.04, and a deactivated periodic boundary in z-direction (to induce bilayer formation in the xy-plane).

**Fig. 10:**
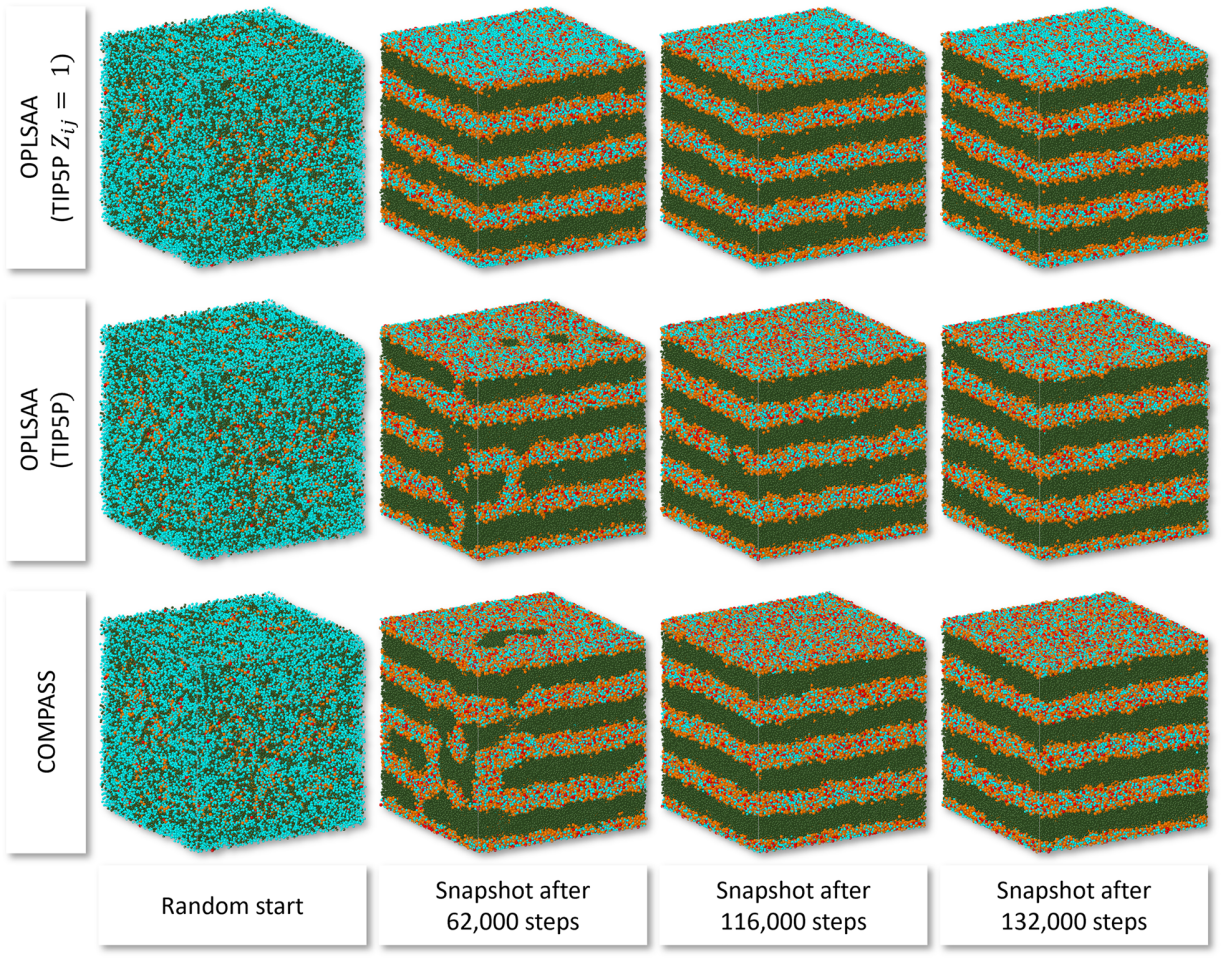
Stacked bilayer superstructure formation from random mixing of a C_10_E_4_/water mixture for particle sets OPLS-AA (TIP5P ${Z}_{ij}=1$) (top row), OPLS-AA (TIP5P) (middle row), and COMPASS (bottom row) with front view of the simulation box with vertical z-axis. Particle colours: Me: Olive, Me2O: Orange, MeOH: Red, H2O: Cyan

**Fig. 11:**
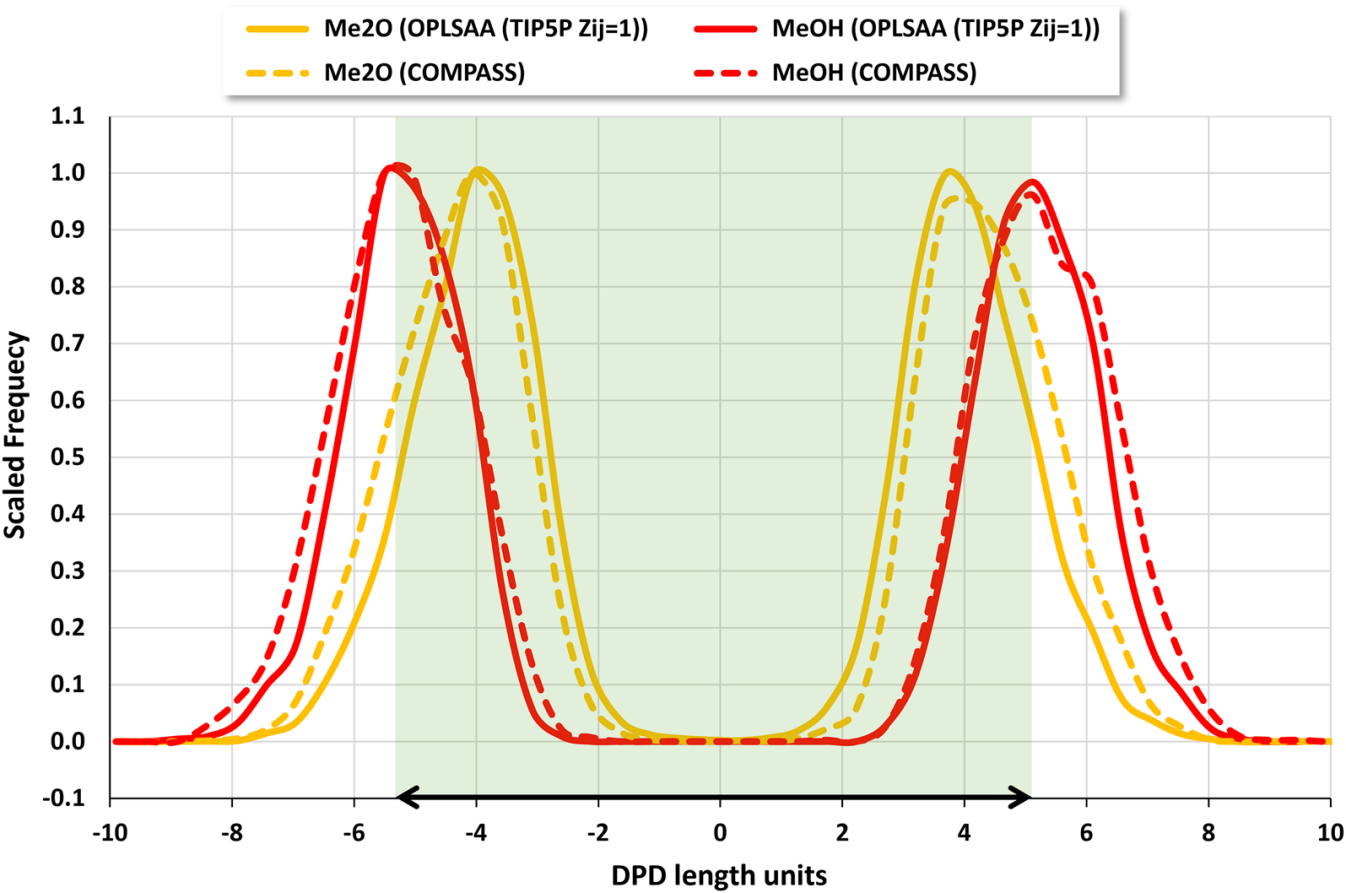
Me2O and MeOH particle distribution snapshots along the z-axis perpendicular to a single emerged C_10_E_4_ bilayer for the OPLS-AA (TIP5P ${Z}_{ij}=1$) (solid lines) and COMPASS (dashed lines) particle sets. The highlighted area corresponds to the bilayer width, indicated by the double arrow

For particle set OPLS-AA (TIP5P ${Z}_{ij}=1$), a stacked bilayer superstructure emerges at simulation step 62,000, see Fig. 10 and Table 4, which was even below the COMPASS particle set from [44] with 116,000 steps, where the emerged bilayer structure corresponds well to the one reported in [44], see Fig. 11. The OPLS-AA (TIP5P) particle set required more than twice as many simulation steps (132,000) and the OPLS-AA (TIP3P) particle set more than tenfold as many (846,000 steps). The AMOEBA09 and MMFF94 particle sets show a bilayer superstructure formation, but the bilayers do not align parallel to the xy-plane (as induced by the periodic boundary conditions) but parallel to the yz-plane. Using the hybrid MM3/MMFF94 particle set, no bilayer was formed within 1,000,000 simulation steps. For the specified DPD simulation task, the OPLS-AA (TIP5P ${Z}_{ij}=1$) particle set can be regarded as the most suitable choice, which is in good agreement with experimental findings.

**Table 4:** Bilayer convergence for different particle sets (with an integration step size of 0.04)

Force field	COMPASS	MMFF94*	MMFF94/MM3	AMOEBA09*	OPLS-AA (TIP3P)	OPLS-AA (TIP5P)	OPLS-AA (TIP5P, ${Z}_{ij}=1$)
Convergence [in 1000 simulation steps]	116	522	–	860	846	132	62

^*^ Bilayer superstructure not parallel to xy-plane

## Conclusions

The outlined automated comprehensive calculation of intermolecular interaction energies based on molecular force-fields shows satisfactory results for small molecule interactions and can even be successfully used to estimate mesoscopic simulation parameters. Special care should always be taken, as individual force fields can lead to erroneous results. Therefore, despite all automation, manual checking of the results is still essential.

The new calculation pipeline can be easily extended to additional force fields (which may require their conversion into the Tinker format). Therefore, calculating differential pair interaction energies will advance with the progress in improving the underlying molecular force fields. Due to the modularized pipeline approach, alternative modeling packages can also be used for certain computational tasks if they can provide the required specific functions.

The robustness of the outlined computational pipeline can be seen as a crucial advance, as the estimation of differential pair interaction energies along alternative computational paths often led to ambiguous results, which in particular prevented the construction of consistent particle–particle repulsions for larger mesoscopic particle sets [47]. The construction of mesoscopic sets with dozens of particles (including hundreds or thousands of mutual particle repulsions) is now within practical reach.

## Availability and requirements

- Project name: Mesoscopic Interaction Parameter Estimation with Tinker for Java (MIPET4Java)
- Project home page: https://github.com/zielesny/MIPET
- Current version: v1.0.0.0
- Operating system(s): Windows (× 64), Linux (× 64)
- Programming language: Java
- Other requirements: Java v17.0.4 or higher, Tinker Molecular Modeling Package v8.10.2
- Licence: GPL-3.0
- Any restrictions to use by non-academics: While the backbone code of this project is not restricted to academic use, the Tinker Molecular Modeling Package is subject to corresponding restrictions, see [21] for license details.

## Data Availability

The source code of the calculation pipeline and the description for downloading the Tinker Molecular Modeling Package are available on GitHub at https://github.com/zielesny/MIPET.

## Abbreviations

AMOEBA09: Atomic Multipole Optimized Energetics for Biomolecular Simulation force field 09
BSSE: Basis set superposition error
COMPASS: Condensed-phase Optimized Molecular Potentials for Atomistic Simulation Studies
CDK: Chemistry Development Kit
DFT: Density Functional Theory
DPD: Dissipative Particle Dynamics
LMOD: Low-mode conformational search
MD: Molecular dynamics
MM3: Molecular mechanics force field 3
MMFF94: Merck molecular force field 94
OCVM: Optimally Conditioned Variable Metric
OPLS-AA: All-atom optimized potentials for liquid simulations force field
RMS: Root mean square
SAPT: Symmetry-Adapted Perturbation Theory
